# Carriers of the m.3243A>G variant should not be labelled with an acronym before they have been systematically screened for multisystem disease

**DOI:** 10.4102/ajlm.v13i1.2527

**Published:** 2024-09-30

**Authors:** Josef Finsterer

**Affiliations:** 1 Neurology and Neurophysiology Centre, Vienna, Austria

We read with interest the article by Makgopa et al. about a 45-year-old male with maternally inherited diabetes and deafness (MIDD) syndrome due to the variant m.3243A>G.^1^ Family history was positive for hearing loss (mother, two sisters, nephew), diabetes (two sisters, nephew), cardiac disease (mother, two sisters), and kidney failure (niece).^1^ Two other family members (third sister, nephew) tested positive for the m.3243A>G variant.^1^ The study is compelling but raises concerns that should be discussed.

The first point is that neither heteroplasmy rates nor mitochondrial DNA (mtDNA) copy number have been determined. In order to assess the rate of progression and outcome, it would have been imperative to at least determine heteroplasmy rates in various affected and unaffected tissues. Since heteroplasmy rates can influence phenotypic presentation and can explain the phenotypic heterogeneity between family members, it would have been helpful to know these values.

The second point is that the cause of heart failure in the index patient and one of his sisters was not specified. Was the heart failure due to coronary artery disease, dilated cardiomyopathy, hypertrophic cardiomyopathy, restrictive cardiomyopathy, Takotsubo syndrome, left ventricular hypertrabeculation, or malignant ventricular arrhythmias? Knowledge of cardiac disease that causes death in at least four family members (mother, index patient, two sisters) is important for the care of other living family members and for genetic counselling. What were the results of long-term electrocardiogram recordings, what were the pro-brain natriuretic peptide values in the clinically affected living family members, and did cardiac magnetic resonance imaging show hypertrophy of the left ventricular myocardium or left ventricular hypertrabeculation? Did the mother have signs of heart failure such as leg oedema, exertional dyspnoea, or neck vein distension before death?

The third point is that we disagree with the diagnosis of MIDD in the index patient. The index patient suffered not only from diabetes and hearing impairment, but also from congestive heart failure, myopathy and renal failure.^1^ Therefore, this patient cannot be diagnosed with MIDD but rather should be classified as MIDD plus.^2^

The fourth point is that the index patient did not undergo autopsy. A patient with a mitochondrial disorder should undergo an autopsy, especially if he or she has not been systematically screened for multisystem involvement throughout his or her lifetime. The m.3243A>G variant may have manifested itself not only in the ears, heart, muscles, and pancreas, but generally in all organs.^3^ Since pulmonary embolism was suspected, it should have been confirmed by autopsy.

Since the m.3243A>G commonly manifests in the brain,^4^ we should know whether any of the clinically affected family members had cognitive decline, seizures, ataxia, movement disorder, or stroke-like episodes.

Surprisingly, the index patient’s mother was depicted as living in the pedigree shown in Figure 2 in Makgopa et al. It should be clarified whether she was deceased as described in the case description or is actually alive as stated in the pedigree.

In conclusion, this interesting study has limitations that put the results and their interpretation into perspective. Removing these limitations could strengthen the conclusions and reinforce the study’s message. All unresolved questions must be clarified before readers can uncritically accept the study’s message. Carriers of the m.3243A>G variant should not be labelled with an acronym before they have been systematically screened for multisystem disease.

## Response from Makgopa et al.

We thank the author of this Scientific Letter for their interest in our article. They have raised some interesting and compelling arguments, and we appreciate their perspective.

However, the title of the letter may be a bit misleading, as it appears to imply that erroneous diagnoses were made in the Makgopa et al. article. Our article sought to highlight the importance of recognising the MIDD pattern and ensuring that maternal relatives of a patient with MIDD are screened and followed up, as they are at risk of developing the condition. Maternal relatives should be tested for the mtDNA variant, as they are likely to be obligate carriers. As stated in our article, genetic testing was considered for available maternal relatives. The article clearly indicates that the proband was not available, as he was deceased (Table 2 in Makgopa et al.) at the time when the genetic testing was conducted.

While testing heteroplasmy may be desirable, the frequency may change with age across tissues, even within the same individual, and the impact of heteroplasmy still remains unresolved. Although not accurate, heteroplasmy levels can be broadly estimated on the restriction fragment length polymorphism band intensities. There is no doubt that these are positive cases. We think it is important to point out that the unavailability of heteroplasmy determination in the local clinical setting cannot be an excuse not to diagnose cases. Capability of an assay to accurately detect mtDNA variants is very important. The polymerase chain reaction–restriction fragment length polymorphism method used in this study has appreciable sensitivity to provide correct diagnosis.

It is interesting that the author suggested the diagnosis of MIDD plus. Indeed, patients described by Makgopa et al. had variable presentations. However, MIDD plus, as suggested in this letter, is not a formal diagnosis. It is noteworthy that none of the patients described by Makgopa et al. were found to have mitochondrial encephalomyopathy with lactic acidosis and stroke-like episodes (MELAS) syndrome.

In conclusion, we hope that concerns raised in the letter do not in any way dilute the important message of the article by Makgopa et al. There is no doubt that the letter further indicates the importance of actively screening patients with MIDD and their maternal relatives, as previously reported. It is worth emphasising that families need to be followed up, as they may be at risk of developing either MIDD, MELAS or other forms of primary mitochondrial disease related to this variant.
